# Statistical Interpretation of Key Comparison Reference Value and Degrees of Equivalence

**DOI:** 10.6028/jres.108.038

**Published:** 2003-12-01

**Authors:** R. N. Kacker, R. U. Datla, A. C. Parr

**Affiliations:** National Institute of Standards and Technology, Gaithersburg, MD 20899-0001, USA

**Keywords:** interlaboratory evaluation, measurement uncertainty, variance components

## Abstract

Key comparisons carried out by the Consultative Committees (CCs) of the International Committee of Weights and Measures (CIPM) or the Bureau International des Poids et Mesures (BIPM) are referred to as CIPM key comparisons. The outputs of a statistical analysis of the data from a CIPM key comparison are the key comparison reference value, the degrees of equivalence, and their associated uncertainties. The BIPM publications do not discuss statistical interpretation of these outputs. We discuss their interpretation under the following three statistical models: nonexistent laboratory-effects model, random laboratory-effects model, and systematic laboratory-effects model.

## 1. Introduction

Key comparisons are interlaboratory comparisons that serve as technical bases for Mutual Recognition Arrangements (MRA) between national metrology institutes (NMIs) [1]. Key comparisons carried out by the Consultative Committees (CCs) of the International Committee of Weights and Measures (CIPM) or the Bureau International des Poids et Mesures (BIPM) are referred to as CIPM key comparisons. Key comparisons carried out by regional metrology organizations (RMO) are referred to as RMO key comparisons. The guidelines for carrying out CIPM key comparisons are given in reference [2].

The objectives of a CIPM key comparison are described in reference [1]. We consider two interpretations of these objectives. A common interpretation is summarized by Nielsen [3] as follows: “The purpose of measurement intercomparisons between NMIs is to test, whether measurements performed in the participating countries are consistent taking into account the uncertainties assigned to the measurements. If an inconsistency is detected, the participating countries should take the corrective actions needed to obtain consistency. Otherwise, measurement results exchanged across borders cannot be considered equivalent without adding a ‘between countries uncertainty,’ which would be in disharmony with the concept of the SI system of units.”

This paper is based on a second interpretation of the objectives of a CIPM key comparison: Generally, the participants of a CIPM key comparison are NMIs that are members of the appropriate Consultative Committee; at least some of these NMIs provide realizations of the SI values to establish the traceability of measurements made in their countries. The purpose of a CIPM key comparison is to establish the key comparison reference value^1^, the degrees of equivalence^2^, and their associated uncertainties on the basis of the data provided by the participants.

This paper is limited to a simple CIPM key comparison where the common measurand is a physical quantity of stable value during the comparison. Many CIPM key comparisons are not simple because it is often impractical or impossible to realize exactly the same measurand for or by all participants. We use the symbol *Y* for the stable value of the measurand. The data provided by the participants of a simple CIPM key comparison are paired results and standard uncertainties [*x*_1_, *u*(*x*_1_)], …, [*x_n_*, *u*(*x_n_*)], where the results *x*_1_, …, *x_n_* are measurements of *Y*. The outputs of a statistical analysis of these data are the key comparison reference value *x*_R_, the degree of equivalence *d_i_* = *x_i_* − *x*_R_ of the result *x_i_*, the degree of equivalence *d_i,j_* = *d_i_* − *d_j_* = *x_i_* − *x_j_* of the results *x_i_* and *x_j_*, and their associated standard uncertainties *u*(*x*_R_), *u*(*d_i_*), and *u*(*d_i,j_*), respectively, for *i, j* = 1, 2, …, *n* and *i* ≠ *j* [1]. The key comparison reference value *x*_R_ is an estimate for *Y.* An estimate for *Y* is a combined result of measurement determined from the data [*x*_1_, *u*(*x*_1_)], …, [*x_n_*, *u*(*x_n_*)].

An understanding of the difference between sampling probability distributions, used in classical (frequentist) statistics, and state-of-knowledge probability distributions, used in Bayesian statistics, is necessary for proper analysis and interpretation of the data from a key comparison. Briefly, they are defined as follows. In classical statistics, the value of the measur- and is assumed to be an unknown constant, often called the true value, and each result of measurement is regarded as a realization of a random variable with a sampling distribution. A *sampling distribution* is a probability distribution that describes the relative frequencies of occurrence for all possible results of measurement when the conditions of measurement are hypothesized to be fixed at the intended levels [4]. The metrologist relates the expected values of the sampling distributions for the results of measurement to the value of the measurand. A classical (frequentist) statistical interpretation is a statement that relates the realized measurements to what one might expect if the key comparison could be repeated infinitely many times and throughout these repetitions the hypothesized sampling distributions continued to apply.

In Bayesian statistics, the measurement data are given constants and the value of the measurand is a random variable. A probability distribution for the value of the measurand is a *state-of-knowledge distribution* that describes the degrees of belief for all possible values that could be attributed to the measurand [4]. The belief is based on all available information including current results of measurement and scientific judgment based on prior and other data. Similar state-of-knowledge distributions apply to the other parameters involved in assessing the value of the measurand. A Bayesian interpretation is a statement that represents the state-of-knowledge about the value of the measurand based on state-of-knowledge distributions before measurements are made and a likelihood function conditional on the current measurements [4]. The ISO *Guide* [5] is consistent with a Bayesian interpretation of measurements but not with a classical (frequentist) interpretation [4].

We refer to the results *x*_1_, …, *x_n_* as laboratory results. The laboratory results *x*_1_, …, *x_n_* are regarded as realizations of random variables *x*_1_, …, *x_n_* with sampling distributions^3^. We use the symbols *X*_1_, …, *X_n_* for the expected values *E*(*x*_1_), …, *E*(*x_n_*) of the sampling distributions of *x*_1_, …, *x_n_*, respectively. We refer to the expected values *X*_1_, …, *X_n_* as the laboratory expected values. We use the symbols *σ*_1_, …, *σ_n_* for the standard deviations *S* (*x*_1_), …, *S* (*x_n_*) of the sampling distributions of *x*_1_, …, *x_n_*, respectively. Here *S*(*x_i_*) is the square root of the variance *V*(*x_i_*) = *E*[*x_i_* − *E*(*x_i_*)]^2^ of the sampling distribution of *x_i_* for *i* = 1, 2, …, *n*. The uncertainties *u*(*x*_1_), …, *u*(*x_n_*) are statistical estimates of *σ*_1_, …, *σ_n_*, respectively.

References [1] and [2] do not discuss statistical interpretations of the pairs [*x*_R_, *u*(*x*_R_)], [*d_i_*, *u*(*d_i_*)], and [*d_i,j_*, *u*(*d_i,j_*)]. A statistical analysis of the data from a key comparison and interpretation of its outputs requires assumptions and models about the relationship between the data [*x*_1_, *u*(*x*_1_)], …, [*x_n_*, *u*(*x_n_*)] and the value *Y* of the measurand. In Sec. 2, we discuss two assumptions, labeled as *Assumption I* and *Assumption II*, about the relationship between the laboratory expected values *X*_1_, …, *X_n_* and *Y*. Then we discuss two classical statistics models, a nonexistent laboratory-effects model and a random laboratory-effects model, based on *Assumption I*. Next, we propose a systematic laboratory-effects model based on *Assumption II.* We describe the key comparison reference value, the degrees of equivalence, and their associated uncertainties determined by each of the three statistical models. In Sec. 3 and 4, we discuss statistical interpretations of the pairs [*x*_R_, *u*(*x*_R_)], [*d_i_*, *u*(*d_i_*)], and [*d_i,j_*, *u*(*d_i,j_*)] under the three statistical models. Our conclusion is given in Sec. 5.

## 2. Statistical Assumptions and Models for the Relationship Between the Data and the Value of the Measurand

In this section, we discuss statistical assumptions and models for analyzing the data from a simple CIPM key comparison to determine the key comparison reference value, the degrees of equivalence, and their associated uncertainties.

### 2.1 Assumptions About the Relationship Between the Laboratory Expected Values and the Value of the Measurand

One may either assume that the laboratory expected values *X*_1_, …, *X_n_* are all equal or allow for the possibility that *X*_1_, …, *X_n_* may not be equal.

*Assumption I*: The expected values *X*_1_, …, *X_n_* are all equal. The *Assumption I* defined so far does not specify the relationship between the results *x*_1_, …, *x_n_* and *Y*. Therefore, in concert with *Assumption I*, it is generally assumed that the common expected value is equal to *Y*, i.e., *X*_1_ = … = *X_n_* = *Y*. Under *Assumption I*, the results *x*_1_, …, *x_n_* are subject to intralaboratory variations only.

*Assumption II*: The expected values *X*_1_, …, *X_n_* may not be equal, i.e., *X_i_ ≠ X_j_* for some *i, j* = 1, 2, …, *n* and *i* ≠ *j*. Therefore, not all of *X*_1_, …, *X_n_* may equal the value *Y* of the measurand. The *Assumption II* defined so far does not specify the relationship between the results *x*_1_, …, *x_n_* and *Y*. Therefore, in concert with *Assumption II*, it is generally assumed that *Y* is either somewhere in the range of results *x*_1_, …, *x_n_* or in the vicinity of this range^4^ [6]. Under *Assumption II*, the results *x*_1_, …, *x_n_* are subject to both the intralaboratory variations represented by the uncertainties *u* (*x*_1_), …, *u* (*x_n_*) and the interlaboratory variation arising from the dispersion of *X*_1_, …, *X_n_* about *Y*. The differences (*X*_1_ − *Y*), …, (*X_n_* − *Y*) are laboratory-effects (biases) due to unrecognized sources of error, denoted by *b*_1_, …, *b_n_*, in the results *x*_1_, …, *x_n_*. The biases are common to all measurements in a particular laboratory but may be different for different laboratories.

### 2.2 Assumption About the Uncertainties Submitted by the Participants

The standard uncertainties *u*(*x*_1_), …, *u*(*x_n_*) submitted by the participants of a key comparison are estimates obtained by combining various estimated components of uncertainty in determining the value *Y* of the measurand. A combined standard uncertainty *u*(*x_i_*) may be unreliable for various reasons. For example, a classical (frequentist) Type A component of *u*(*x_i_*) calculated from a small number of independent measurements is unreliable^5^ [5]. A Type A component of *u*(*x_i_*) based on unjustified statistical assumptions may be unreliable. A Type B component of *u*(*x_i_*) based on unreasonable state-of-knowledge distributions may be unreliable. A combined uncertainty *u*(*x_i_*) determined from an incomplete measurement equation may be an underestimate. The unreliability of estimated uncertainties *u*(*x*_1_), …, *u*(*x_n_*) is a component of uncertainty in determining the key comparison reference value *x*_R_, the degrees of equivalence *d_i_* and *d_i,j_*, and their associated standard uncertainties. In this paper, we do not discuss the additional uncertainty that arises from the unreliability of *u*(*x*_1_), …, *u*(*x_n_*).

Classical (frequentist) statistical analyses and interpretations discussed in this paper are based on the assumption that the estimated uncertainties *u*(*x*_1_), …, *u*(*x_n_*) are equal to the true standard deviations *σ*_1_, …, *σ_n_* of the sampling distributions of *x*_1_, …, *x_n_*, respectively. Most metrologists make this assumption. For example, the expression *u*(*x*_W_) = 1/√[Σ*_i_ w_i_*] for the standard deviation of the weighted mean *x*_W_ = Σ*_i_ w_i_ x_i_* / Σ*_i_ w_i_*, where *w_i_* = 1/*u*^2^(*x_i_*) for *i* = 1, 2, …, *n*, requires this assumption.

Statistical analyses based on the ISO *Guide* regard a laboratory expected value *X_i_* as a variable with a state-of-knowledge distribution having expected value *x_i_* and standard deviation *u*(*x_i_*). Such analyses require the assumption that the estimated uncertainties *u*(*x*_1_), …, *u*(*x_n_*) are sufficiently reliable.

### 2.3 Classical (Frequentist) Statistics Models Based on *Assumption I*

The weighted mean *x*_W_ = Σ*_i_ w_i_ x_i_* / Σ*_i_ w_i_* and the expression *u*(*x*_W_) = 1/√[Σ*_i_ w_i_*], where *w_i_* = 1/*u*^2^(*x_i_*) for *i* = 1, 2, …, *n*, are often used as the key comparison reference value *x*_R_ and its associated standard uncertainty *u*(*x*_R_), respectively. The use of *x*_W_ as *x*_R_ and *u*(*x*_W_) as *u*(*x*_R_) is based on the following classical (frequentist) statistics model.

#### 2.3.1 Nonexistent Laboratory-Effects Model

The results are regarded as realizations of the random variables *x*_1_, …, *x_n_*, where

$$x_{i}=Y+e_{i},$$ (1)

and *e_i_* = (*x_i_* − *Y*) is the error in *x_i_* for *i* = 1, 2, …, *n*. In this model, the parameter *Y* is identified with the value of the measurand and the errors *e*_1_, …, *e_n_* are mutually independently distributed random variables with sampling distributions. The sampling distributions of *e*_1_, …, *e_n_* are generally assumed to be normal (Gaussian). The expected values of *e*_1_, …, *e_n_* are assumed to be zero and the variances of *e*_1_, …, *e_n_* are assumed to be *u*^2^(*x*_1_), …, *u*^2^(*x_n_*), respectively. Under model (1) [represented by Eq. (1)], the expected value *E*(*x_i_*) is equal to *Y* and the variance *V*(*x_i_*) is equal to *u*^2^(*x_i_*), for *i* = 1, 2, …, *n*. Since the expected values of all results are equal to *Y*, the model (1) is based on *Assumption I*. In model (1), the results *x*_1_, …, *x_n_* are free of laboratory-effects (biases). Therefore, we refer to it as a nonexistent laboratory-effects model. The best least-squares estimate for the parameter *Y* of the nonexistent laboratory-effects model (1) is the weighted mean *x*_W_ = Σ*_i_ w_i_ x_i_*/Σ*_i_ w_i_*, where *w_i_* = 1/*u*^2^(*x_i_*) for *i* = 1, 2, …, *n*. The term best least-squares estimate^6^ means that the estimate *x*_W_ has the smallest variance among all estimates of *Y* that are both linear functions of the results *x*_1_, …, *x_n_* and have the expected value *Y*. The standard deviation of the sampling distribution of *x*_W_ is *u*(*x*_W_) = 1/√[Σ*_i_ w_i_*]. Thus the key comparison reference value *x*_R_ based on model (1) is *x*_W_ and *u*(*x*_R_) is *u*(*x*_W_). The corresponding degrees of equivalence are *d_i_* = *x_i_* − *x*_W_ and *d_i,j_* = *x_i_* − *x_j_*, for *i*, *j* = 1, 2, …, *n* and *i* ≠ *j*. The uncertainties *u*(*d_i_*) and *u*(*d_i,j_*) are determined from the sampling distributions of *x*_1_, …, *x_n_* and *x*_R_ under model (1).

*Note* 1: When not all uncertainties *u*(*x*_1_), …, *u*(*x_n_*) are sufficiently reliable estimates of the true standard deviations *σ*_1_, …, *σ_n_*, the true standard deviation of the sampling distribution of the weighted mean *x*_W_ may be larger than the true standard deviation of the sampling distribution of the arithmetic mean *x*_A_. Thus in this case the weighted mean *x*_W_ may be an inferior key comparison reference value to the arithmetic mean *x*_A_.

#### 2.3.2 Random Laboratory-Effects Model

The classical statistics model based on *Assumption I* for the situation where the dispersion of results *x*_1_, …, *x_n_* may be more than what can reasonably be attributed to the intralaboratory variances *u*^2^(*x*_1_), …, *u*^2^(*x_n_*) is as follows. The results are regarded as realizations of the random variables *x*_1_, …, *x_n_*, where

$$x_{i}=Y+b_{i}+e_{i},$$ (2)

*b_i_* = (*X_i_* − *Y*) is the laboratory effect (bias) in *x_i_* and *e_i_* = (*x_i_* − *X_i_*) is the intralaboratory error in *x_i_* for *i* = 1, 2, …, *n*. The classical statistics assumptions to relate the results *x*_1_, …, *x_n_* to *Y* are as follows: the laboratory biases *b*_1_, …, *b_n_* are regarded as random variables having the same normal sampling distribution with expected value zero and variance *σ*_b_^2^ ≥ 0, called *interlaboratory variance*; and *b*_1_, …, *b_n_* are assumed to be mutually independent and independent of the errors *e*_1_, …, *e_n_*. The model (2) [represented by Eq. (2)] with these assumptions is referred to as a random laboratory-effects model [7]. Here the term random means that the biases *b*_1_,…,*b_n_* are regarded as random variables with the same sampling distribution that is assumed to be normal with expected value zero and variance *σ*_b_^2^. Under the random laboratory-effects model (2), the expected value *E*(*x_i_*) is equal to *Y* and the variance *V*(*x_i_*) is equal to *σ*_b_^2^ + *u*^2^(*x_i_*) for *i* = 1, 2, …, *n*. The nonexistent laboratory-effects model (1) is a special case of the random laboratory-effects model (2) where *σ*_b_^2^ = 0, which means that the biases *b*_1_, …, *b_n_* are all zero, i.e., *X*_1_ = … = *X_n_* = *Y*.

A popular estimate for the parameter *Y* of model (2) is the weighted mean^7^ *x*_W_ = Σ*_i_ w_i_ x_i_* / Σ*_i_ w_i_*, where *w_i_* = 1/[*s*_b_^2^ + *u*^2^ (*x_i_*)] and *s*_b_^2^ is an estimate for *σ*_b_^2^. Reference [8] discusses various methods for determining *s*_b_^2^. The estimate *s*_b_^2^ inflates each of the intralaboratory variances *u*^2^(*x*_1_), …, *u*^2^(*x_n_*) just enough to account for the dispersion of results *x*_1_, …, *x_n_* that is not accounted for by model (1). Under the assumption that the estimated variances *s*_b_^2^ + *u*^2^ (*x*_1_), …, *s*_b_^2^ + *u*^2^(*x_n_*) are regarded as the true variances of the sampling distributions of *x*_1_, …, *x_n_*, the best estimate of the parameter *Y* of model (2) is the weighted mean *x*_W_ and the standard uncertainty associated with *x*_W_ is *u*(*x*_W_) = 1/√[Σ*_i_ w_i_*], where *w_i_* = 1/[*s*_b_^2^ + *u*^2^(*x_i_*)] for *i* = 1, 2, …, *n* [9], [8]. Thus the key comparison reference value *x*_R_ based on model (2) is the weighted mean *x*_W_ = Σ*_i_ w_i_ x_i_* / Σ*_i_ w_i_* and uncertainty *u*(*x*_R_) is *u*(*x*_W_) = 1/√[Σ*_i_ w_i_*], where *w_i_* = 1/[*s*_b_^2^ + *u*^2^(*x_i_*)] for *i* = 1, 2, …, *n*. The corresponding degrees of equivalence are *d_i_* = *x_i_* − *x*_R_ = *x_i_* − *x*_W_ and *d_i,j_* = *x_i_* − *x_j_*, for *i*, *j* = 1, 2, …, *n* and *i* ≠ *j*. The uncertainties associated with the degrees of equivalence are determined from the sampling distributions of *x*_1_, …, *x_n_* and *x*_R_ under model (2).

The advantage of model (2) relative to model (1) is that it allows for the possibility that the dispersion of results *x*_1_, …, *x_n_* may be more than what can reasonably be attributed to the intralaboratory variances *u*^2^(*x*_1_), …, *u*^2^(*x_n_*). When the dispersion of *x*_1_, …, *x_n_* is not more than what can reasonably be attributed to *u*^2^(*x*_1_), …, *u*^2^(*x_n_*), the estimate *s*_b_^2^ is zero. In that case, model (2) yields the same *x*_R_ and *u*(*x*_R_) as model (1). Therefore, there is no disadvantage to using model (2) in place of model (1).

The random laboratory-effects model (2) of classical statistics is conceptually faulty for the analysis of a CIPM key comparison for the following reasons. First, the participants of a CIPM key comparison are specific NMI laboratories rather than randomly chosen from a large population of laboratories. Therefore, the biases *b*_1_, …, *b_n_* may not be regarded as random variables with the same sampling distribution. Second, the assumption that the sampling distribution of the biases *b*_1_, …, *b_n_* is a normal distribution with expected value zero may not be justified. The next section introduces a new model that does not assume that the biases *b*_1_, …, *b_n_* are random variables with a normal sampling distribution.

### 2.4 A Model Based on *Assumption II* and the ISO *Guide*

A statistical analysis of the data from a simple CIPM key comparison based on *Assumption II* requires one to account for the uncertainty that arises from the unknown bias in a combined result of measurement that is used as an estimate for *Y*. Before publication of the ISO *Guide*, there was no generally accepted approach to account for the uncertainty that arises from an unknown bias. The approach proposed by the ISO *Guide* to account for the uncertainty that arises from an unknown bias is now generally accepted. So we have used the ISO *Guide* to develop the following systematic laboratory-effects model.

#### 2.4.1 Systematic Laboratory-Effects Model

We start with a combined result of the form ∑*_i_ a_i_ x_i_*, where Σ*_i_ a_i_* = 1, that is used as an initial estimate for *Y*. This estimate requires the assumption that *Y* is within the range of results *x*_1_, …, *x_n_*. We refer to the initial estimate as the uncorrected combined result (UCR) and denote it by *x*_UCR_ = Σ*_i_ a_i_ x_i_*. If *a_i_* = *w_i_* / Σ*_i_ w_i_*, then *x*_UCR_ is the weighted mean *x*_W_ = Σ*_i_ w_i_ x_i_* / Σ*_i_ w_i_*, where *w* = 1/*u*^2^(*x_i_*) for *i* = 1, 2, …, *n*. If *a_i_* = 1/*n* for *i* = 1, 2, …, *n*, then *x*_UCR_ is the arithmetic mean *x*_A_ = Σ*_i_ x_i_* / *n*. Let *X*_UCR_ = Σ*_i_ a_i_ X_i_* be the expected value of the sampling distribution of *x*_UCR_. According to *Assumption II*, the result *x*_UCR_ is subject to the bias (*X*_UCR_ − *Y*). The ISO *Guide* recommends that the result *x*_UCR_ should be corrected to counter its possible bias and the uncertainty associated with the correction should be included in the combined standard uncertainty associated with the corrected result. The bias (*X*_UCR_ − *Y*) is an unknown constant but the correction for bias, denoted by *C*, is a variable with a state-of-knowledge probability distribution. If the expected value and standard deviation of a state-of-knowledge probability distribution for the correction variable *C* are denoted by *c* and *u*(*c*), respectively, then the correction applied to the result *x*_UCR_ to counter its possible bias is *c* and the standard uncertainty associated with the correction is *u*(*c*).

In order to specify a state-of-knowledge probability distribution for the correction variable *C*, the laboratory expected values *X*_1_, …, *X_n_* and the value *Y* of the measurand are regarded as variables with state-of-knowledge distributions and the data *x*_1_, …, *x_n_* and *u*(*x*_1_), …, *u*(*x_n_*) are regarded as given constants. A state-of-knowledge distribution for *X_i_* represents the state of knowledge about the value *Y* of the measurand in the laboratory labeled *i* for *i* = 1, 2, …, *n*. The expected value *E*(*X_i_*) and standard deviation *S*(*X_i_*) of the variable *X_i_* are assumed to be *x_i_* and *u*(*x_i_*), respectively, for *i* = 1, 2, …, *n* [5], [4]. It follows that *X*_UCR_ = Σ*_i_ a_i_ X_i_* is a variable with a state-of-knowledge probability distribution. The expected value of *X*_UCR_ is *E*(*X*_UCR_) = Σ*_i_ a_i_ E*(*X_i_*) = Σ*_i_ a_i_ x_i_* = *x*_UCR_. In the expression (*Y* − *X*_UCR_) for the negative of bias, treated as a variable, we replace *X*_UCR_ with its expected value *x*_UCR_. Then a probability distribution for *C* represents belief about the possible values of (*Y* − *x*_UCR_), where *x*_UCR_ is a constant and *Y* is the variable. The belief about possible values of *Y* is based on all available information including results of measurement and scientific judgment. In reference [6], we proposed a triangular distribution for the correction variable *C*, with peak at 0 and default limits [*x*_(1)_ − *x*_UCR_] = min{*x*_1_ − *x*_UCR_, …, *x_n_* − *x*_UCR_} and [*x*_(_*_n_*_)_ − *x*_UCR_] = max{*x*_1_ − *x*_UCR_, …, *x_n_* − *x*_UCR_}. A criticism of the proposed triangular distribution with default limits is that it is determined by the extreme results *x*_(1)_ = min{*x*_1_, …, *x_n_*} and *x*_(_*_n_*_)_ = max{*x*_1_, …, *x_n_*}, which are sometimes suspected to be in error.

Here, we propose a discrete-equal-probability distribution that is determined by all of the results *x*_1_, …, *x_n_*. The results *x*_1_, …, *x_n_* are plausible values of *Y* as determined by competent laboratories.^8^ So the known constant differences (*x*_1_ − *x*_UCR_), …, (*x_n_* − *x*_UCR_) are plausible values of (*Y* − *x*_UCR_). These differences are a statistical basis for specifying a probability distribution for *C*. Let *c_i_* = *x_i_* − *x*_UCR_ for *i* = 1, 2, …, *n*. Suppose *c*_1_, …, *c_n_* are assigned probabilities *p*_1_, …, *p_n_*. Then the expected value of *C* is *c* = *E*(*C*) = Σ*_i_ p_i_ c_i_* = (Σ*_i_ p_i_ x_i_*) − *x*_UCR_ and the standard deviation of *C* is *u*(*c*) = *S*(*C*) = √[Σ*_i_p_i_* (*c_i_* − *c*)^2^]. Frequently, the available scientific knowledge is inadequate to assign different probabilities *p*_1_, …, *p_n_* to *c*_1_, …, *c_n_*. Therefore, we propose the discrete-equal-probability distribution for which *p_i_* = 1/*n* for *i* = 1, 2, …, *n*. The expected value and standard deviation of *C* based on discrete-equal-probability distribution are *c* = *x*_A_ − *x*_UCR_ and *u*(*c*) = √[Σ*_i_* (*x_i_* − *x*_A_)^2^/*n*], respectively, where *x*_A_ = Σ*_i_ x_i_* / *n* is the arithmetic mean of the results *x*_1_, …, *x_n_*.

A measurement equation is required to incorporate correction for possible bias in a combined result of measurement for *Y*. The measurement equation that corresponds to the bias (*X*_UCR_ − *Y*) in the uncorrected combined result *x*_UCR_ is *Y* = *X*_UCR_ + *C*. This measurement equation is widely applicable in metrology [10]. It suggests the following model for the value *Y* of the measurand:

$$E\left(X_{i}\right)=x_{i},S\left(X_{i}\right)=u\left(x_{i}\right),X_{\text{UCR}}=\sum_{i}a_{i}X_{i},Y=X_{\text{UCR}}+C,$$ (3)

where *a*_1_, …, *a_n_* are constants such that Σ*_i_ a_i_* = 1. In this model, *X*_1_, …, *X_n_*, *X*_UCR_, *C*, and *Y* are variables with state-of-knowledge distributions. The expected value and standard deviation of *X_i_* are the given constants *x_i_* and *u*(*x_i_*), respectively, for *i* = 1, 2, …, *n*. A state-of-knowledge distribution for the correction variable *C* is defined independently of the state-of-knowledge distributions for the variables *X*_1_, …, *X_n_*, after the latter have been specified. In particular, *X*_UCR_ and *C* are independently distributed. We refer to model (3) [represented by Eq. (3)] as a systematic laboratory-effects model to distinguish it from the random laboratory-effects model (2) that regards the biases (systematic errors) *b*_1_, …, *b_n_* as random variables having the same sampling distribution with expected value zero. Suppose the standard deviation of the variable *X*_UCR_ is *S*(*X*_UCR_) = *u*(*x*_UCR_). Then the corrected combined result for *Y* determined from the systematic laboratory-effects model (3) is *y* = *x*_UCR_ + *c* and its associated standard uncertainty is *u*(*y*) = √[*u*^2^(*x*_UCR_) + *u*^2^(*c*)].

The systematic laboratory-effects model (3) allows for the possibility that not all pairs of the variables *X*_1_, …, *X_n_* may be independently distributed. The variance *V*(*X*_UCR_) = *u*^2^(*x*_UCR_) is determined from the variances and covariances of the variables *X*_1_, …, *X_n_*. When the distributions of *X*_1_, …, *X_n_* are independent and *X*_UCR_ is the weighed mean *X*_W_ = Σ*_i_ w_i_ X_i_* / Σ*_i_ w_i_*, where *w_i_* = 1/*V*(*X_i_*) = 1/*u*^2^(*x_i_*) for *i* = 1, 2, …, *n*, then *u*^2^(*x*_UCR_) = *V*(*X*_W_) = 1/[Σ*_i_ w_i_*] = 1/{Σ*_i_* [1/*u*^2^(*x_i_*)]. When the distributions of *X*_1_, …, *X_n_* are independent and *X*_UCR_ is the arithmetic mean *X*_A_ = Σ*_i_ X_i_* / *n*, then *u*^2^(*x*_UCR_) = *V*(*X*_A_) = (1/*n*^2^)Σ*_i_ V*(*X_i_*) = (1/*n*^2^)Σ*_i_ u*^2^(*x_i_*)^9^.

In order to specify *c* and *u*(*c*), one is free to use any reasonable distribution for *C*, based on scientific judgment. When the discrete-equal-probability distribution is used, *c* = *x*_A_ − *x*_UCR_ and *u*(*c*) = √[Σ*_i_* (*x_i_* − *x*_A_)^2^/*n*]. In that case, the result of measurement for *Y* is *y* = *x*_UCR_ + *c* = *x*_UCR_ + *x*_A_ − *x*_UCR_ = *x*_A_ and *u*(*y*) = √[*u*^2^(*x*_UCR_) + *u*^2^(*c*)], where *u*(*c*) = √[Σ*_i_* (*x_i_* − *x*_A_)^2^/*n*].

Following the ISO *Guide*, the result *y* and uncertainty *u*(*y*) determined from the systematic laboratory-effects model (3) are interpreted as the expected value and standard deviation of a state-of-knowledge distribution for the values that could reasonably be attributed to *Y* based on the data *x*_1_, …, *x_n_* and *u*(*x*_1_), …, *u*(*x_n_*) [5], [4], [6]. Thus the key comparison reference value *x*_R_ based on the systematic laboratory-effects model (3) is *y* and uncertainty *u*(*x*_R_) is *u*(*y*). The corresponding degrees of equivalence are *d_i_* = *x_i_* − *y* and *d_i,j_* = *x_i_* − *x_j_* for *i*, *j* = 1, 2, …, *n* and *i* ≠ *j*. The uncertainties *u*(*d_i_*) and *u*(*d_i,j_*) are determined from state-of-knowledge distributions for the variables *X*_1_, …, *X_n_* and *Y*.

## 3. Interpretation of the Key Comparison Reference Value and Its Associated Uncertainty

### 3.1 Classical Statistics Models Based on *Assumption I*

The nonexistent laboratory-effects model and the random laboratory-effects model are based on classical (frequentist) statistics. In particular, the results *x*_1_, …, *x_n_* are regarded as realizations of random variables with sampling distributions and *Y* is an unknown constant. Therefore, the key comparison reference value *x*_R_ is a realization of a random variable with a sampling distribution that has expected value *Y* and standard deviation *u*(*x*_R_) = *u*(*x*_W_) = 1/√[Σ*_i_ w_i_*]. In the nonexistent laboratory-effects model *w_i_* is 1/*u*^2^(*x_i_*) and in the random laboratory-effects model *w_i_* is 1/[*s*_b_^2^ + *u*^2^(*x_i_*)] for *i* = 1, 2, …, *n*. The interval [*x*_R_ ± 2*u*(*x*_R_)] determined from a classical statistics model is a confidence interval for *Y* computed from the data *x*_1_, …, *x_n_* and *u*(*x*_1_), …, *u*(*x_n_*). Imagine that the CIPM key comparison could be repeated infinitely many times in exactly the same conditions using exactly the same instruments and artifacts. Now imagine that throughout these repetitions exactly the same sampling distributions continued to apply to the random variables *x*_1_, …, *x_n_*. Then the confidence level is the fraction of the infinitely many hypothetical intervals, such as [*x*_R_ ± 2*u*(*x*_R_)], that would include *Y* [4].

### 3.2 Systematic Laboratory-Effects Model Based on *Assumption II*

The key comparison reference value *x*_R_ and uncertainty *u*(*x*_R_) determined from the systematic laboratory-effects model are given constants that represent the expected value and standard deviation of a state-of-knowledge distribution for *Y* based on the data *x*_1_, …, *x_n_* and *u*(*x*_1_), …, *u*(*x_n_*). The interval [*x*_R_ ± 2*u*(*x*_R_)] determined from the systematic laboratory-effects model is an expanded uncertainty interval for *Y*. The coverage probability (level of confidence) of the interval [*x*_R_ ± 2*u*(*x*_R_)] is the fraction of a state-of-knowledge distribution for *Y* that is encompassed by this interval [4].

## 4. Interpretation of the Degrees of Equivalence and Their Associated Uncertainties

### 4.1 Classical Statistics Models Based on *Assumption I*

In the random laboratory-effects model and its special case the nonexistent laboratory-effects model, the expected values of the sampling distributions of *x*_1_, …, *x_n_*, and *x*_R_ are all equal to *Y*. Therefore, the expected values of the sampling distributions of all degrees of equivalence *d_i_* = *x_i_* − *x*_R_ and *d_i,j_* = *x_i_* − *x_j_* are zero, for *i*, *j* = 1, 2, …, *n* and *i* ≠ *j*. This implies that all computed degrees of equivalence, whether small or large, are statistical estimates of zero. In particular, according to these models, all degrees of equivalence published in the key comparison database (KCDB) [11] are estimates of zero.

### 4.2 Systematic Laboratory-Effects Model Based on *Assumption II*

In the systematic laboratory-effects model, the results *x*_1_, …, *x_n_* are the expected values and the uncertainties *u*(*x*_1_), …, *u*(*x_n_*) are the standard deviations of state-of-knowledge distributions for the laboratory expected values *X*_1_, …, *X_n_*, treated as variables. It follows that the degree of equivalence *d_i_* = *x_i_* − *x*_R_ = *x_i_* − *y* is the expected value of a state-of-knowledge distribution for the laboratory effect (bias) *X_i_* − *Y* for *i* = 1, 2, …, *n*, and the degree of equivalence *d_i,j_* = *x_i_* − *x_j_* is the expected value of a state-of-knowledge distribution for the difference *X_i_* − *X_j_* for *i*, *j* = 1, 2, …, *n* and *i* ≠ *j*. The uncertainty *u*(*d_i_*) is the standard deviation^10^ of *X_i_* − *Y* and the uncertainty *u*(*d_i,j_*) is the standard deviation of *X_i_* − *X_j_*, for *i*, *j* = 1, 2, …, *n* and *i* ≠ *j*.

## 5. Conclusion

We addressed a simple CIPM key comparison where the common measurand is a physical quantity of stable value during the comparison. We discussed statistical interpretation of the key comparison reference value, the degrees of equivalence, and their associated uncertainties determined from the following three statistical models: nonexistent laboratory-effects model, random laboratory-effects model, and systematic laboratory-effects model. The first two models are based on classical (frequentist) interpretation of measurements. The systematic laboratory-effects model is based on Bayesian interpretation of measurements.

The key comparison reference value *x*_R_ and uncertainty *u*(*x*_R_) determined from the systematic laboratory-effects model represent the expected value and standard deviation of a state-of-knowledge distribution for the value *Y* of the measurand. Therefore their statistical interpretation agrees with the ISO *Guide*. According to the systematic laboratory-effects model, the degree of equivalence *d_i_* and uncertainty *u*(*d_i_*) are, respectively, the expected value and standard deviation of a state-of-knowledge distribution for the laboratory effect (bias) *X_i_* − *Y*, for *i* = 1, 2, …, *n*, and the degree of equivalence *d_i,j_* and uncertainty *u*(*d_i,j_*) are, respectively, the expected value and standard deviation of a state-of-knowledge distribution for the difference *X_i_* − *X_j_*, for *i*, *j* = 1, 2, …, *n* and *i* ≠ *j*. Thus the degrees of equivalence determined from the systematic laboratory-effects model quantitate the agreements and disagreements of laboratory results. Therefore, the systematic laboratory-effects model is suitable for the data analysis of a simple CIPM key comparison.

## Footnotes

1 “Key comparison reference value: the reference value accompanied by its uncertainty resulting from a CIPM key comparison [1].”

2 “Degree of equivalence of a measurement standard: the degree to which the value of a measurement standard is consistent with the key comparison reference value. This is expressed quantitatively by the deviation from the key comparison reference value and the uncertainty of this deviation. The degree of equivalence between two measurement standards is expressed as the difference between their respective deviations from the key comparison reference value and the uncertainty of this difference [1].”

3 We use the symbols *x*_1_, …, *x_n_* for both the random variables and their realized values.

4 If the expected value *X*_1_ were equal to the value *Y* of the measurand, then according to the ISO *Guide*, the interval [*x*_1_ ± 2*u*(*x*_1_)] would represent an approximate range of the plausible values of *Y*. Likewise, if *X*_2_ were equal to *Y* then the interval [*x*_2_ ± 2*u*(*x*_2_)] would represent an approximate range of the plausible values of *Y*, and so on for *X*_3_, *X*_4_, …, *X_n_*. It follows from *Assumption II* that any one or more of the expected values *X*_1_, …, *X_n_* may be close to or equal to *Y*; therefore, the total interval consisting of the union of intervals [*x_i_* ± 2*u*(*x_i_*)], for *i* = 1, 2, …, *n*, represents an approximate range of the plausible values of *Y*. However, most metrologists assign greater belief-probability to the middle than to the ends of the total interval.

5 The unreliability of a classical (frequentist) estimate of uncertainty arising from a small number of measurements is quantified by degrees of freedom [5].

6 Least-squares estimation does not require that the errors *e*_1_, …, *e_n_* and hence *x*_1_, …, *x_n_* have normal distributions.

7 We did not introduce a new symbol for the weighted mean determined from model (2) because model (1) is a special case of model (2).

8 As noted in the footnote of Sec. 2.1, the total interval consisting of the union of intervals [*x_i_* ± 2*u*(*x_i_*)], for *i* = 1, 2, …, *n*, represents an approximate range of the plausible values of *Y*.

9 Since the harmonic mean of positive numbers is less than or equal to their arithmetic mean, *V*(*X*_W_) ≤ *V*(*X*_A_). When *u*^2^(*x*_1_), … , *u*^2^(*x_n_*) are equal, *V*(*X*_W_) = *V*(*X*_A_).

10 The standard deviation of *X_i_* − *Y* depends on the covariance between *X_i_* and *Y* for *i* = 1, 2, …, *n*. Since *Y* = *X*_UCR_ + *C* = Σ*_i_ a_i_ X_i_* + *C* and the variable *C* is distributed independently of the variables *X*_1_, …, *X_n_*, the covariances *C*(*X_i_*, *Y*), for *i* = 1, 2, …, *n*, can be determined from the variances and covariances of *X*_1_, …, *X_n_*. Then *u*(*d_i_*) = √[*V*(*X_i_* − *Y*)], where the variance *V*(*X_i_* − *Y*) is equal to *V*(*X_i_*) + *V*(*Y*) − 2×*C*(*X_i_*, *Y*).
