# Erratum: Statistical Interpretation of Key Comparison Reference Value and Degrees of Equivalence

**DOI:** 10.6028/jres.109.020

**Published:** 2004-04-01

**Authors:** R. N. Kacker, R. U. Datla, A. C. Parr

**Affiliations:** National Institute of Standards and Technology, Gaithersburg, MD 20899-0001, USA

[J. Res. Natl. Inst. Stand. Technol. Volume 108, Number 6, November–December 2003, p. 439]

On page 444, line 24 in the left column should be “the correction variable *C*, with peak at 0 and default”

